# Molar pregnancy with normal viable fetus presenting with severe pre-eclampsia: a case report

**DOI:** 10.1186/s13256-018-1689-9

**Published:** 2018-05-21

**Authors:** Freddie Anak Atuk, Juliana Binti Mohamad Basuni

**Affiliations:** Department of Obstetrics & Gynecology Miri General Hospital, Sarawak, Malaysia

**Keywords:** Partial molar, Normal viable fetus, Pre-eclampsia

## Abstract

**Background:**

While gestational trophoblastic disease is not rare, hydatidiform mole with a coexistent live fetus is a very rare condition occurring in 0.005 to 0.01% of all pregnancies. As a result of the rarity of this condition, diagnosis, management, and monitoring will remain challenging especially in places with limited resources and expertise. The case we report is an interesting rare case which presented with well-described complications; only a few similar cases have been described to date.

**Case presentation:**

We report a case of a 21-year-old local Sarawakian woman with partial molar pregnancy who presented with severe pre-eclampsia in which the baby was morphologically normal, delivered prematurely, and there was a single large placenta showing molar changes.

**Conclusion:**

Even though the incidence of this condition is very rare, recognizing and diagnosing it is very important for patient care and it should be considered and looked for in patients presenting with pre-eclampsia.

## Background

Molar pregnancy is significantly more common in extremes of age [1]. Hydatidiform mole has been recognized as a clinical entity since the time of Hippocrates and has always aroused interest because of its wide spectrum of presentations and rare spectacular complications [2]. Asian countries show the highest rates, followed by Africa and Latin America whereas Europe, Australia, and the USA generally report the lowest rates [3].

Most pregnancies in which molar change has been reported in association with a normal fetus represent a dizygotic twin pregnancy with one complete hydatidiform mole and other normal twin with clearly distinguishable molar regions in the placenta [4]. The incidence of a normal live fetus and a partial molar placenta such as the case we describe is extremely rare.

## Case presentation

**Fig. 1:**
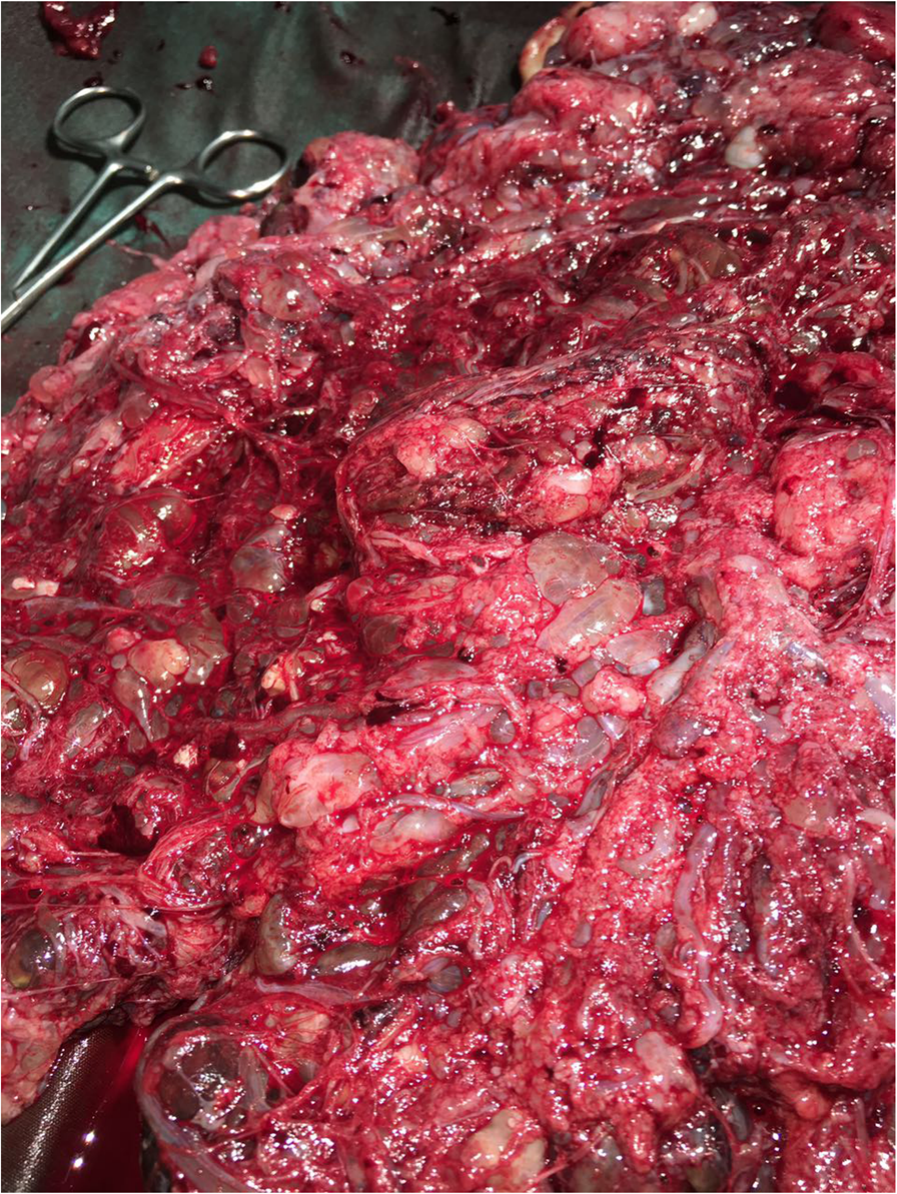
Single large placenta with diffuse cystic changes

**Fig. 2:**
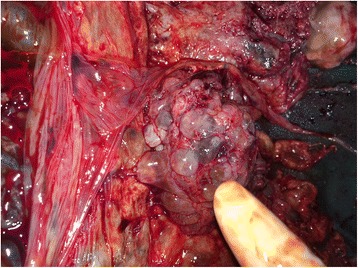
Single large placenta with diffuse cystic changes

A 21-year-old local Sarawakian primigravida woman was diagnosed as having severe pre-eclampsia at 28 weeks and was admitted for blood pressure stabilization and monitoring. On assessment, her fundal height was larger than indicated by date and transabdominal ultrasound scans, which was suggestive of molar changes in the placenta with a viable fetus noted. She went into spontaneous labor a few days later and lower segment caesarean section was done for breech presentation. A grossly normal baby girl weighing 990 g was delivered. Unfortunately, the baby died due to complications of prematurity and sepsis on day 12 of life. The placenta was noted to be large with diffuse cystic changes (Figs. 1 and 2). Pathological study showed placental tissue weighed 2300 g measuring 280 × 230 × 70 mm and it was friable with many vesicles of variable sizes ranging from 10 to 12 mm. The histopathological finding was compatible with partial molar pregnancy. Our patient is currently doing well on regular follow-up and her beta-human chorionic gonadotropin (hCG) was normal 1 month after delivery.

## Discussion

Partial molar pregnancy coexisting with normal live fetus as seen in our case is an extremely rare condition excluding cases of multiple conceptions. Such an association has been divided into three types: The first and most common is a twin pregnancy with a normal fetus having a normal placenta and a complete mole; the second type is a twin pregnancy with a normal fetus and placenta and a partial mole; and the third and most uncommon occurrence is a singleton normal fetus with partial molar placenta. In cases of a singleton normal fetus with partial molar placenta, the fetus must have a normal karyotype to survive *in utero*, although its placenta can have some variation, from diploidy of the amnion to triploidy of the chorionic villi [4].

Molar pregnancy with coexisting fetus carries a significant risk to both mother and the fetus. Maternal risks include abnormal bleeding, pre-eclampsia, eclampsia, hyperthyroidism, anemia, persistent gestational trophoblastic disease, preterm delivery, and abruption [1]. According to Vejerslev’s review on 113 reports of pregnancies with mole and fetus in which there appeared to be no major malformations or cytogenetic abnormalities, of the 87 who had intended to continue the pregnancy with or without knowledge of accompanying mole, 52 pregnancies (59.8%) proceeded to the 28th week, and a risk for either substantial bleeding or pre-eclamptic symptoms developed in approximately 30% [5]. On the other hand, fetal complications include abortion, congenital anomalies, preterm, intrauterine growth restriction, and intrauterine fetal death [1]. In the case we describe, both the mother and baby were affected by those complications namely pre-eclampsia and preterm birth.

Diagnosis will remain a challenge as this condition is very rare which makes it less likely to be suspected in women with pre-eclampsia. Early diagnosis or detection of this condition might not happen in places where detailed ultrasound screening is not a routine for all pregnant women due to lack of facilities and trained sonographer.

Management of molar changes associated with normal-appearing fetus varies and still remains challenging and debatable. The decision to continue the pregnancy will largely depend on the presence or absence of complications to the mother or baby, prior obstetric history, as well as the woman’s wishes after adequate counselling. Post-delivery, the patient needs to be put on close follow-up. The initial hCG value at delivery should be registered and weekly values plotted on a standard regression curve adjusted for local reference standards. This is followed by measuring weekly values until three values are obtained below the detection limit, then every second week for 2 months, and then monthly for 1 year after the first negative value [6].

## Conclusions

Even though a case of partial molar with coexisting normal fetus is a very rare occurrence, it carries a very high risk to the mother and fetus. One of its classic well-described complications or presentation is pre-eclampsia, such as the case we presented. Therefore, a patient who presents with pre-eclampsia needs to be assessed thoroughly not only looking for its severity and complications but also the possible underlying problem, such as a coexisting molar pregnancy.
